# Experimental and Theoretical Analysis of Rayleigh and Leaky-Sezawa Waves Propagating in ZnO/Fused Silica Substrates

**DOI:** 10.3390/mi15080974

**Published:** 2024-07-29

**Authors:** Cinzia Caliendo, Massimiliano Benetti, Domenico Cannatà, Farouk Laidoudi, Gaetana Petrone

**Affiliations:** 1 Institute for Photonics and Nanotechnologies, IFN-CNR, Via del Fosso del Cavaliere 100, 00133 Rome, Italy; 2 Institute of Microelectronics and Microsystems, IMM-CNR, Via del Fosso del Cavaliere 100, 00133 Rome, Italy; massimiliano.benetti@cnr.it (M.B.); domenico.cannata@cnr.it (D.C.); gaetana.petrone@artov.imm.cnr.it (G.P.); 3 Research Center in Industrial Technologies CRTI, P.O. Box 64, Cheraga 16014, Algiers, Algeria; f.laidoudi19@gmail.com; 4 Department of Astronautical, Electrical and Energy Engineering of the University of Rome “La Sapienza”, Via Eudossiana 18, 00184 Rome, Italy

**Keywords:** ZnO, fused silica, Rayleigh wave, split finger IDTs, Sezawa wave, FEM study, propagation loss, K^2^

## Abstract

Piezoelectric c-axis oriented zinc oxide (ZnO) thin films, from 1.8 up to 6.6 µm thick, have been grown by the radio frequency magnetron sputtering technique onto fused silica substrates. A delay line consisting of two interdigital transducers (IDTs) with wavelength λ = 80 µm was photolithographically implemented onto the surface of the ZnO layers. Due to the IDTs’ split-finger configuration and metallization ratio (0.5), the propagation of the fundamental, third, and ninth harmonic Rayleigh waves is excited; also, three leaky surface acoustic waves (SAWs) were detected travelling at a velocity close to that of the longitudinal bulk wave in SiO_2_. The acoustic waves’ propagation in ZnO/fused silica was simulated by using the 2D finite-element method (FEM) technique to identify the nature of the experimentally detected waves. It turned out that, in addition to the fundamental and harmonic Rayleigh waves, high-frequency leaky surface waves are also excited by the harmonic wavelengths; such modes are identified as Sezawa waves under the cut-off, hereafter named leaky Sezawa (LS). The velocities of all the modes was found to be in good agreement with the theoretically calculated values. The existence of a low-loss region in the attenuation vs. layer thickness curve for the Sezawa wave below the cut-off was theoretically predicted and experimentally assessed.

## 1. Introduction

Rayleigh waves are surface acoustic waves (SAWs) that travel along the surface of elastic half-spaces. If the propagating medium is piezoelectric, the SAW propagation can be excited by electrical means and is characterized by both mechanical displacement components and an electrical potential wave, predominantly concentrated up to about one wavelength in depth from the propagating medium surface. When the propagating medium consists in a piezoelectric film/substrate heterostructure, multiple Rayleigh modes can propagate [1,2] if the velocity of the transverse bulk acoustic wave (BAW) in the substrate is higher than that in the overlayer. This heterostructure is named a *slow on fast* structure and the ZnO/fused silica combination is a typical example since the shear BAW velocities in fused silica and ZnO are equal to 3766 and 2829 m/s, respectively. All the high-order modes except the fundamental wave have a cut-off frequency: below the cut-off, the velocity of the modes is higher than the transverse BAW velocity in the substrate and thus they leak energy in the substrate; above the cut-off, the velocity of the modes decreases steadily with frequency and tends to the shear velocity of the BAW in the layer material. As a result, the leaky region below the cut-off is characterized by a non-zero attenuation, as opposed to the region above the cut off where the waves travel poorly attenuated. In the available scientific literature [3,4,5,6,7], the propagation of Sezawa waves below the cut-off, i.e., leaky Sezawa waves (LS), has been observed experimentally and predicted theoretically in structures consisting of a highly anisotropic fast half-space (such as 4H-SiC, diamond, and sapphire) covered by a thin epitaxial piezoelectric layer (such as GaN, ScAlN, or AlN). Like GaN, AlN, and ScAlN, zinc oxide is also a piezoelectric material which can be grown in the form of a highly oriented thin film; its SAW velocity is slower than that of the formers, but it has the largest electromechanical coupling coefficient K^2^ among them. Since the leaky Sezawa modes maintain the K^2^ of the piezoelectric overlayer, the use of a low-cost substrate (such as fused silica) covered by a ZnO thin layer represents a remarkable advantage in view of reducing the fabrication costs while ensuring high performances.

This paper reports numerical simulations and experimental results on the generation of Rayleigh and leaky-Sezawa waves in low-cost ZnO/fused silica substrates. A delay line consisting of two split-finger interdigital transducers (IDTs) with a metallization ratio of 0.5 and wavelength λ = 80 µm was photolithographically implemented onto the surface of the ZnO layer. The propagation of fundamental and even harmonic Rayleigh waves is excited in the ZnO/SiO_2_ substrate in the ~37 to ~300 MHz frequency range, namely the fundamental, third, and ninth harmonic SAWs, for a ZnO layer thickness ranging from 1.8 up to 6.6 µm. Leaky SAWs were also detected in the ~72 to ~500 MHz frequency range. The SAWs’ propagation in ZnO/SiO_2_ was simulated using the two-dimensional (2D) finite-element method (FEM) technique (by the commercial software COMSOL Multiphysics 6.1) to identify the nature of the experimentally detected waves. It turns out that, in addition to the fundamental and harmonic Rayleigh waves, leaky surface waves are also excited by the harmonic wavelengths: such high-frequency modes are identified as Sezawa waves below the cut-off. The FEM analysis was performed to study the modes’ characteristics (modes’ shape, velocity, electroacoustic coupling efficiency K^2^, and propagation loss) and to assign each experimentally observed frequency the proper SAW mode. The experimental data were found in good accordance with the theoretically predicted values. The existence of a low-loss region for the Sezawa wave below the cut-off was theoretically predicted and experimentally assessed.

Exploiting split-finger IDTs, which operate highly efficiently at the third harmonic wave, to excite high-velocity high-K^2^ LS modes in ZnO/SiO_2_ substrates allows us to achieve an improved resonant frequency by using electrodes with finger- and inter-finger spacing easily achievable with inexpensive conventional photolithographic techniques [8].

The LS modes are worthy of investigation because of their K^2^ and velocity larger than the Rayleigh wave which leads to enhanced operation frequency of the SAW devices without requiring expensive nanofabrication methods to reduce the metal electrode widths.

## 2. Materials and Methods

### 2.1. SAW Propagation in Layered Media

Surface acoustic waves can travel along the surface of an elastic half-space with a phase velocity which depends on the crystallographic anisotropy of the propagating medium. In single-material half-space, the surface trapping of the acoustic waves originates from the existence of a stress-free surface (i.e., the half-space/air interface). The propagation of the SAWs along the surface of a piezoelectric medium is excited and detected by metal interdigitated transducers (IDTs) which consist of a series of thin-film metallic electrodes with periodic arrangement, positioned onto the medium surface; the fingers’ periodicity represents the wavelength λ of the travelling acoustic mode. By applying an alternating voltage to the launching IDT, a periodic electric field is imposed on the piezoelectric substrate’s surface and a SAW is produced because of the generation of a periodic strain field. The generated SAW travels through a certain distance (the wave path) and reaches the output IDT which detects the electric voltage associated with the SAW. The IDTs’ parameters (such as the IDT finger width and spacing, fingers overlapping, and the number of finger pairs) affect the IDTs’ performance; the double electrode split IDT structure minimizes internal reflections (they are called *non-reflective transducers* because the internal reflection phenomenon is shifted at the double of the center frequency) that otherwise complicate the IDT response [9,10,11,12]. The ability of the split IDT to generate harmonics of the fundamental mode strongly depends on the metallization ratio of the fingers [13,14]: for a 0.5 metallization ratio, the split-finger IDT allows for highly efficient excitation of the third harmonic and hence high working frequency ranges which would otherwise require expensive lithographic techniques; the fifth and seventh harmonics are suppressed as the wave has the same electric potential at all IDT fingers.

If the propagating medium consists of a layer/substrate structure, multiple SAWs can propagate if the transverse BAW velocity of the half-space material is greater than that of the layer material. The fundamental mode is named Rayleigh wave while the higher-order modes are called Sezawa, R3, R4, and so on [1]. The number of propagating SAW modes and their velocities depend on the layer thickness, h: for a very thin film thickness (h/λ << 1), only the fundamental Rayleigh mode propagates with a velocity close to the SAW velocity of the substrate material (about 3450 m/s for fused quartz). By increasing the layer thickness (h/λ >> 1), the Rayleigh velocity decreases and asymptotically reaches the SAW velocity of the layer material (2644 m/s for c-ZnO), while the velocity of the higher-order modes asymptotically reaches the velocity of the shear BAW in the layer material.

The amplitude profile of the Rayleigh wave is mostly trapped in the layer as opposed to that of the higher-order modes which have an exponential tail in the substrate. The latter modes are characterized by a layer thickness-to-wavelength cut-off at which the phase velocity is equal to the shear BAW velocity of the substrate. At the cut-off, the higher-order modes couple with the bulk modes: the acoustic power of the modes flows into the bulk substrate, thus resulting in a large insertion loss (IL).

### 2.2. FEM Analysis

**Figure 1:**
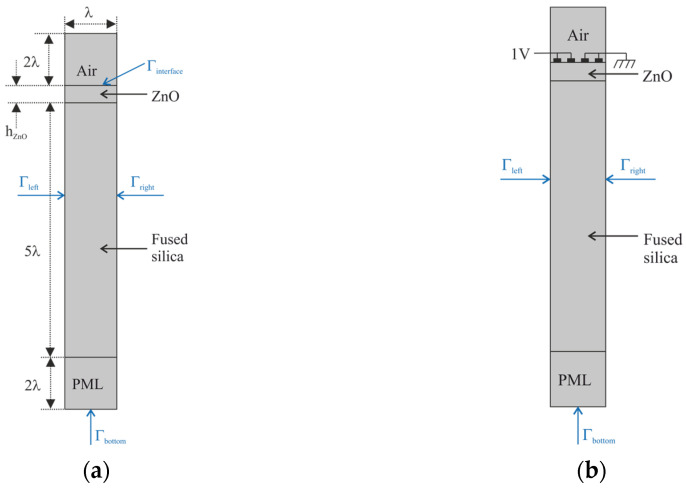
(**a**) The unit cell (not in scale) consisting of four domains: air (2·λ thick), ZnO (from 1 to 80 µm thick), SiO_2_ (5·λ thick), and PML (2·λ thick); (**b**) the unit cell including the Al electrodes.

The propagation of SAWs along the ZnO/SiO_2_ structure was investigated by using 2D FEM analysis because, due to the crystallographic symmetry of the c-ZnO and the isotropy of the fused silica substrate, the acoustic field was unchanged along the transverse direction. Comsol Multiphysics 6.1 software was used in the eigenfrequency and frequency domain studies. The models use a piezoelectric multiphysics coupling node with solid mechanics and electrostatic interfaces. The unit cell (width λ = 80 µm) consists of the following domains, as shown in Figure 1a: air (2·λ thick), ZnO (from 1 to 80 µm thick), SiO_2_ (5·λ thick), and a perfectly matched layer (PML) (2·λ thick). Four Al electrodes (0.15 µm thick and λ/8 wide) were placed onto the surface of the ZnO layer to perform the frequency domain study, as shown in Figure 1b: two neighboring electrodes were connected to a fixed electric potential (terminal type: voltage, 1 V) and the other two were grounded. Periodic boundary conditions ($\Gamma_{left}$ and $\Gamma_{right}$) are applied to the lateral sides of the unit cell. The bottom boundary is fixed ($\Gamma_{bottom}$), while an electrically free or shorted boundary condition on the ZnO free surface ($\Gamma_{interface}$) is used to calculate the electromechanical coupling coefficient (as explained later).

The material constants were extracted from the material library of Comsol. The ZnO has a 0.01 permittivity loss and 0.002 mechanical loss; SiO_2_ has an isotropic mechanical loss equal to 0.01. An extremely fine mesh (physics-defined triangular elements automatically generated by Comsol) was selected for all the FEM simulations.

**Figure 2:**
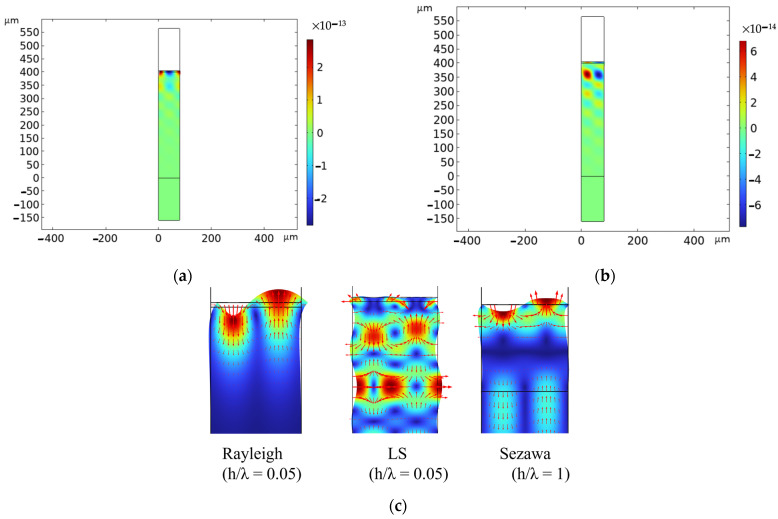
(**a**) The longitudinal and (**b**) the shear displacement component of the leaky Sezawa wave. (**c**) The displacement field (arrows) of the Rayleigh and LS at h/λ = 0.05 and of the Sezawa mode at h/λ = 1.

Eigenfrequency and frequency domain studies were performed to identify the type of the acoustic modes experimentally detected based on a comparison of the experimental data with numerical calculations. Figure 2a,b show, as an example, the longitudinal and shear displacement components (u and v) of the LS wave for a ZnO layer that is 4 µm thick (h/λ = 0.05); from the figure, it can be noticed that the u component is larger than the v component, and the former is also more concentrated inside the layer than the latter, which penetrates inside the SiO_2_ substrate. Figure 2c shows the displacement field (arrows) of the Rayleigh and the LS at h/λ = 0.05 and of the Sezawa at h/λ = 1: the picture highlights the elliptical polarization of the Rayleigh wave, the predominantly shear vertical polarization of the Sezawa wave, and the predominantly longitudinal polarization of the LS wave.

The fundamental and harmonic Rayleigh waves have elliptical polarization and travel along the surface of the propagating medium, up to a depth of about 1.5·λ from the surface. The LS waves are mostly longitudinally polarized far below the cut-off, and their vertical displacement component increases while approaching the cut-off. The LS waves leak energy from the layer into the substrate and become evanescent, thus exhibiting their leaky nature as demonstrated by the substantial vertical displacement component penetrating into the SiO_2_ substrate.

**Figure 3:**
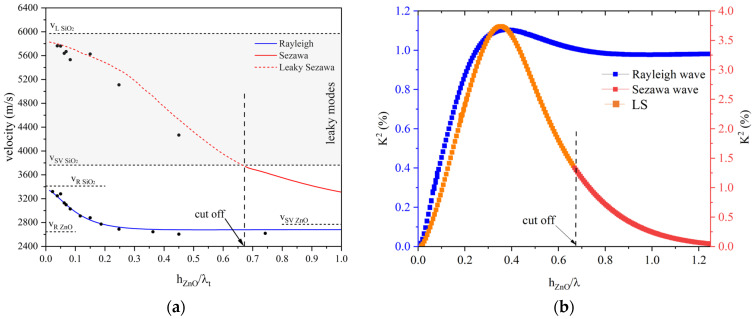
(**a**) The theoretical phase velocity dispersion curves of the Rayleigh, Sezawa, and LS modes (blue, solid red, and dotted red, respectively) travelling in the ZnO/SiO_2_ structure; the black points refer to the experimental measures. (**b**) The theoretical K^2^ vs. ZnO h/λ curves of the Rayleigh and Sezawa modes travelling in the ZnO/SiO_2_ structure.

The phase velocities of the SAWs were calculated for different ZnO layer thicknesses and a fixed wavelength (λ = 80 µm) by performing the sweep parameter eigenfrequency study: the modes’ velocity was calculated as v = λ*f*, with λ = 80 µm being the wavelength and *f* the eigenfrequency solution. Figure 3a shows the Rayleigh, Sezawa, and LS dispersion curves (solid blue, solid red, and dotted red, respectively); the black points are the experimentally measured data which will be discussed in the next paragraphs.

For very thin film thicknesses, only the Rayleigh mode propagates; the Sezawa mode appears as it exceeds its cut-off value (${\left.\frac{h}{\lambda }\right|}_{cut\;off}$ = 0.68). With further increases in h/λ (not shown in Figure 3), several unattenuated higher-order Rayleigh modes appear at a succession of values of ${\left.\frac{h}{\lambda }\right|}_{cut\;off}$. Above the transverse BAW velocity of the substrate, no modes are allowed to propagate unattenuated: by assuming non-zero dielectric loss and mechanical damping, the velocity dispersion curves of the Sezawa and higher-order mode extend between the transverse and longitudinal BAW velocity in the substrate material; in this velocity range, these waves become leaky modes, amplified through coupling with the bulk modes of the substrate, in which they radiate. At small h/λ, the LS velocity is close to that of the longitudinal BAW in SiO_2_, while with the high h/λ limit, it tends to the velocity of the shear vertical BAW in the ZnO.

The electromechanical coupling coefficient K^2^, which is a measure of the electrical-to-acoustic energy conversion efficiency by electrical means, was evaluated from eigenfrequency analysis by calculating the phase velocity of the modes with an electrically free (*v_f_*) and shorted (*v_s_*) boundary condition onto the ZnO free surface ($\Gamma_{interface}$) and using the following approximate formula: $K^{2}=2\cdot \frac{v_{f}-v_{s}}{v_{f}}$. Figure 3b shows the K^2^ dispersion curves for the Rayleigh and Sezawa waves: the K^2^ dispersion curves for the Sezawa (red curve) and LS (orange curve) waves are distinguished based on the colors used. The Sezawa wave is less surfacy than the Rayleigh wave as it travels inside both the substrate and the layer. It has a very low K^2^ and high propagation loss for very small h/λ since the acoustic penetration depth extends beyond the film/substrate interface. From Figure 3b, it can be noticed that, below the cut-off ${\left.\frac{h}{\lambda }\right|}_{cut\;off}$, the K^2^ of the LS increases and reaches a peak (about 3.7%) at h/λ = 0.375; as h/λ decreases further, the K^2^ reaches zero because the ZnO layer thins and α increases. For h/λ larger than the cut-off, the K^2^ tends to zero and the loss asymptotically reaches the loss in the substrate since the Sezawa wave is mostly confined in the SiO_2_ substrate which is non-piezoelectric and lossy.

**Figure 4:**
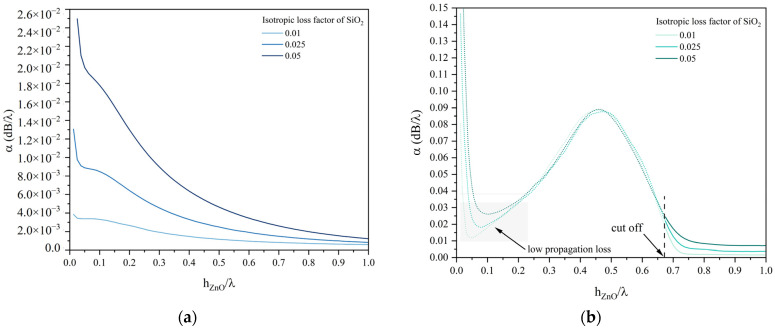
(**a**) The propagation loss dispersion curves for the Rayleigh wave. (**b**) The propagation loss dispersion curves for the Sezawa and LS waves (distinguished by solid and dashed lines). The isotropic loss factor of the SiO_2_ substrate is the running parameter.

The admittance Y vs. frequency curves were calculated by performing the frequency domain study: the waves’ propagation loss was calculated by applying the formula $\alpha =\frac{\omega }{2Qv_{g}}$, where ω=2πf, $v_{g}=v_{ph}+\frac{h}{\lambda }\frac{\partial v_{ph}}{\partial \frac{h}{\lambda }}$ is the group velocity, $v_{ph}$ is the phase velocity, and $Q=\frac{f_{r}}{{∆f}_{3dB}}$ [15]; $f_{r}$ and ${∆f}_{3dB}$ were evaluated from the real part of the Y vs. frequency curve as the abscissa of the peak and as the range of frequencies covered by the half-power bandwidth. Figure 4a,b show the propagation loss dispersion curves for the Rayleigh and Sezawa waves for different values of the isotropic loss factor tanδ_SiO2_ of the SiO_2_ substrate. In Figure 4b, the shading (gray colored) onto the dotted α curve of the Sezawa mode highlights the portion of the curve corresponding to the propagation of low-loss LS waves.

The α dispersion curves of the Rayleigh wave (Figure 4a) were calculated for the SiO_2_ isotropic loss factor tanδ_SiO2_ equal to 0.01, 0.025 and 0.05: for h/λ << 1, the Rayleigh wave attenuation α changes by one order of magnitude (from about 10^−3^ to about 10^−2^ dB/λ), while for h/λ approaching 1, the α curves merge, which is consistent with the surface character of the wave.

At very low h/λ ratios, the penetration depth of the Rayleigh wave extends beyond the interface between the substrate and the thin film, making the substrate crucial for losses. By increasing the layer thickness, the Rayleigh wave experiences a decreasing propagation loss and an increasing K^2^, which asymptotically reaches that of the ZnO half-space while the propagation loss tends to zero since the wave travels predominantly along the ZnO layer, thus it becomes unaffected by the presence of the lossy substrate (the wave suffers only the loss from the layer).

In Figure 4b, the portion of the α dispersion curves before the cut-off has been dashed to highlight the distinction between the Sezawa wave (solid lines) and LS wave (dashed lines); the different colors of the curves refer to the different values of the isotropic loss factor tanδ_SiO2_ of the SiO_2_ substrate. Within the low propagation loss region (i.e., the gray colored portion of the α curve), the LS wave is mostly longitudinally polarized and its K^2^ is lower than it is in the area with high losses and predominant vertical polarization, near the cut-off.

The Sezawa wave is affected by the SiO_2_ loss since the wave travels into the SiO_2_ substrate more than the Rayleigh wave does. The effects of energy leakage of the LS mode are shown in Figure 4b: above the cut-off, the Sezawa wave is affected by the SiO_2_ loss factor since it propagates inside both the layer and the substrate; below the cut-off, the energy leakage into the bulk dominates, thus the resulting LS propagation loss is poorly affected by the choice of the substrate damping. Inside the low-loss region, the LS mode behaves like the Sezawa wave above the cut-off: as a result, the wave propagation loss is affected by the substrate damping.

The LS mode appears in a h/λ region where the Rayleigh mode is also excited: the two waves are easily distinguishable in the S_21_ spectrum since they have quite different frequencies and good K^2^. In the h/λ >> 1 range where higher-order modes asymptotically reach the velocity of the shear BAW in the layer material, multiple modes are excited whose operating frequencies are very close each other and their K^2^ is low; as a result, the modes are difficult to distinguish because the frequency responses are partially overlapped.

Higher-order Rayleigh waves differ from the fundamental wave because the former have the energy distributed between the layer and the substrate, while the latter has the energy concentrated on the top surface of the layer. This has the advantage that the propagation characteristics, such as velocity and attenuation, of higher-order modes are less affected by surface contamination and are less sensitive to changes in the acoustic and/or electrical conditions of the surface.

From the calculations carried out, it turned out that the Sezawa mode is characterized by a dispersion of leaky waves: the cut-off frequency is not a discrete point in the phase velocity vs. h/λ curves but is a long-period termination of a wave surface that approaches the bulk longitudinal velocity of the substrate with increasing propagation loss except in a small low-loss region whose existence is confirmed by the measured data. Since the low-loss conditions for LS waves propagating in the ZnO/SiO_2_ structure can approach a velocity of 5840 m/s (equal to almost double the Rayleigh wave velocity), operation frequencies of several hundreds of MHz are achievable, which are suitable to fabricate high-frequency SAW resonators and sensors.

## 3. Experimental Section

### 3.1. SAW Device Fabrication

Piezoelectric c-axis oriented zinc oxide (ZnO) thin films, from about 1.8 up to 6.6 µm thick, have been grown by radio frequency (RF) magnetron sputtering (by Ionvac Process s.r.l.) onto fused silica and Si substrates in the same sputtering run. The films were sputtered from a 4″ diameter high-purity (99.999%) zinc target, at 200 °C, in an Ar/O_2_ mixture (ratio 1:4) at a pressure of 3.7 × 10^−3^ Torr, 200 W rf power.

**Figure 5:**
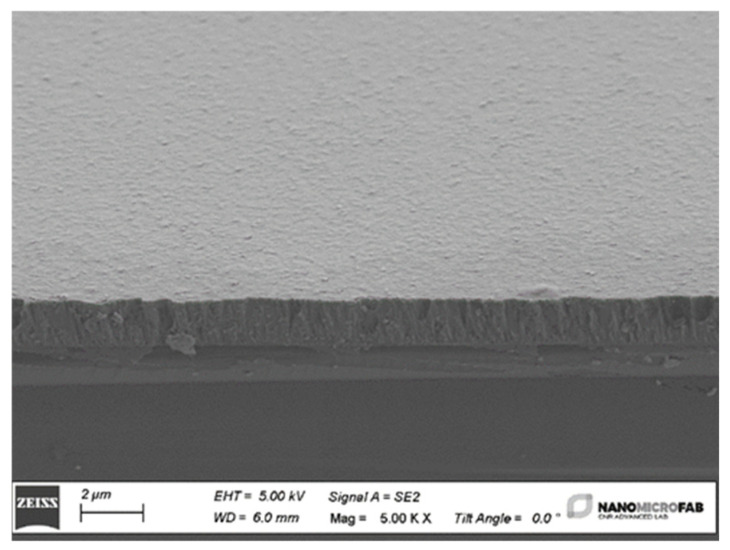
The SEM photo of the surface and cross section of the ZnO/Si structure.

The surface micrographs of the ZnO thin films with various thicknesses were recorded using a scanning electron microscope (SEM) Zeiss Evo MA10 and allowed for the estimation of the ZnO deposition rate (13.3 nm/min). A representative image of a 4 µm thick ZnO thin film, shown in Figure 5, depicts a smooth surface and a columnar structure. The picture highlights both the surface and the cross section of the film and refers to the ZnO film grown onto Si because the latter can be cut more conveniently than fused silica for cross-sectional imaging. The ZnO films were c-axis oriented [16], uniform, and highly adhesive to the substrate.

**Figure 6:**
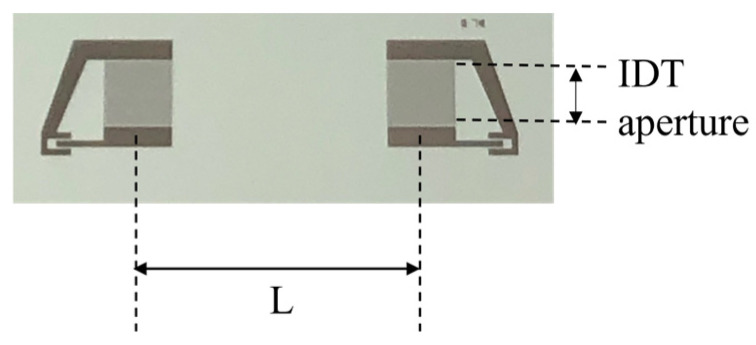
The picture of the SAW delay line implemented onto ZnO/fused silica substrate.

SAW delay lines were photolithographically implemented onto the free surface of the ZnO layer by the conventional optical lithographic technique and lift-off process. The delay lines consist of a couple of Al IDTs: each IDT has 80 electrodes in split-finger configuration to minimize the wave reflection, with an electrode width of 10 μm and a periodicity of λ = 80 μm. The IDT fingers’ overlapping length is 1568 μm and the IDT’s center-to-center distance is L = 6600 μm, as shown in Figure 6. The SAW delay lines were attached to a printed circuit board (PCB) and the solder pads of the IDTs were electrically connected to the SMA connectors to test their frequency response.

### 3.2. SAW Device Test

**Figure 7:**
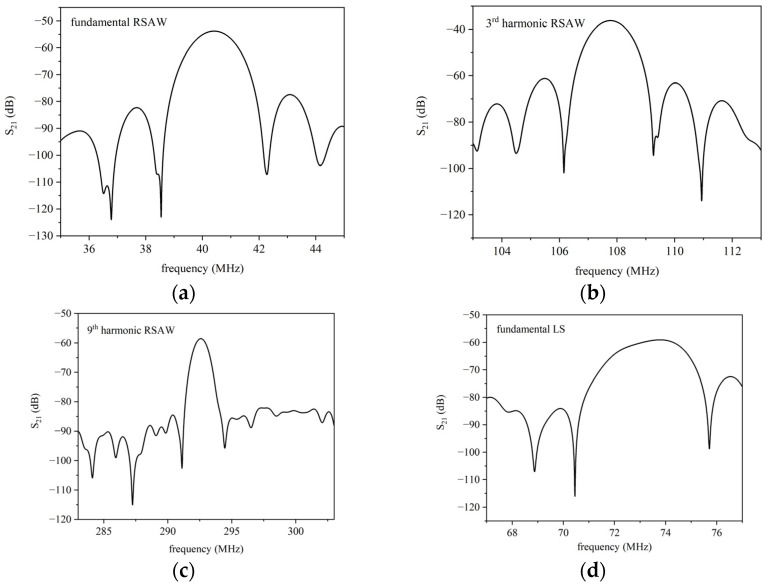

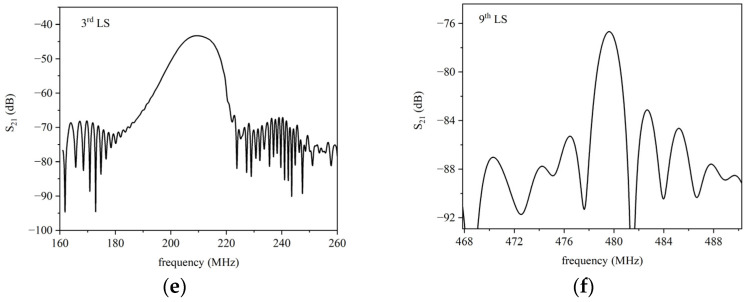
The frequencies corresponding to the surface modes experimentally detected in ZnO (4 µm)/SiO_2_ are the following: (**a**) 40.41, (**b**) 107.75, (**c**) 292.6, (**d**) 73.5, (**e**) 209.70, and (**f**) 479.64 MHz.

The scattering parameters of the SAW delay lines were tested by means of a vector network analyzer (DG8SAQ VNWA 3 from SDR Kits) in the dark, at a controlled humidity (40%) and temperature (24 °C). Figure 7a–f show, as an example, the S_21_ vs. frequency curves of six modes detected in the bilayer structure, for ZnO film that is 4 µm thick. The measurements were performed with the VNA calibrated (full two ports’ calibration) with SOLT kit (SOLT = Short, Open, Load, Through) up to the coaxial cables. The calibration of the VNA excludes the test fixture which consists of two SMA connectors mounted onto the PCB where the ZnO/fused silica substrate is fixed, and the IDT pads are electrically connected to the SMA connectors by Al wires soldered by ultrasonic bonding. All the curves in Figure 7a–f were obtained by using time gating configured to gate out the spurious time signals: the gate center was placed at the peak of the time response and the gate span was adjusted to include all the sidelobes; then, the inverse transform of the gated data was performed to obtain the idealized frequency response.

Several SAW delay lines were tested for different ZnO thicknesses (1.83, 3.1, 4.0, 4.98, and 6.6 µm). The frequencies of the fundamental and harmonic modes were measured for both the Rayleigh and LS waves. The experimental modes’ velocity values, $v_{ph}=f\cdot \lambda$, were obtained by multiplying the operating frequency *f* by the corresponding wavelength; the estimated velocities were plotted inside the graph of Figure 3.

## 4. Discussion

The propagation of LS waves has been experimentally observed and theoretically predicted in heterostructures comprising a piezoelectric layer and a highly anisotropic substrate such as SiC, diamond, LiNbO_3_, and sapphire. Table 1 lists some examples of bi-layered structures where the Sezawa waves were observed above the transonic state. The large variety of examples cited in the table, which also includes non-piezoelectric materials, demonstrates that LS waves can be found in both high- and low-symmetry layered structures.

The experimental data listed in Table 1 demonstrate the following key features:

1. Among the listed structures, the ZnO/SiO_2_ stands out for its technological ease and the low cost of the materials. Indeed, the reactive RF magnetron sputtering technique is a well-established, high-deposition-rate, and relatively low-temperature (T = 200 °C) process which is compatible with standard microelectronic technology. Since the sputtered ZnO films are highly c-axis oriented, uniform, and extremely adhesive to the substrate, they can be a valid alternative to thin epitaxial piezoelectric layers deposited using techniques that require expensive and dedicated equipment.
2. Fused silica, unlike expensive and highly anisotropic substrates, is a low-cost, optically transparent substrate: its high resistance to chemicals, low coefficient of thermal expansion, and excellent optical qualities make it attractive for applications in optics operating in the deep UV and visible wavelength range [16,25,26].
3. The value of the losses that can be read from the S_21_ curves of Figure 6 basically comprise 6 dB from IDTs’ bidirectionality loss (attributed to the SAW propagation toward both sides of each IDT), miscellaneous losses (due to electrical finger resistance and IDT electrical mismatch with the peripheral circuits), and acoustic propagation loss. The largest contribution to the overall loss, the electrical mismatch of the IDTs with the characteristic impedance of the VNA, comes from using a non-optimized SAW delay line geometry implemented on the tested devices. This geometry is general-purpose and not specifically designed for ZnO/SiO_2_-based devices. By optimizing the IDT parameters (the directivity and the number of finger pairs) on the K^2^ dispersion curve and possibly adopting a tuning circuit to match the VNA load impedance to the input and output ports, the frequency response of the devices based on LS can be improved. Moreover, a different IDT design can be considered, such as unidirectional interdigital transducers (UITs) which are optimized to release most of the SAW energy in one preferred direction, floating electrode unidirectional IDT (FEUDT) [27], or single phase-unidirectional IDT (SPUDT), which can suppress the triple-crossing echo signal and reduce the insertion loss of the device due to its inherent unidirectionality [28,29], to cite just a few examples.

Reference [30] is a review paper on SAW devices based on the propagation of the Sezawa wave: many types of heterostructures are described (such as ZnO/SiO_2_/Si, ZnO/Si, ScAlN/diamond, ScAlN/Si, AlN/SiO_2_/Si, ZnO/sapphire, AlN/SiC, AlN/diamond, etc.) and remarkable properties are underlined such as a phase velocity and K^2^ which are higher than those of the Rayleigh wave for an equal thickness of the piezoelectric layer and the same IDT design. Nevertheless, the reference mentions the technological difficulty of growing ZnO layers thick enough to generate the Sezawa mode with a wavelength of about 10 µm. And this is where the presently studied LS comes into play, which, in a particular thickness range and for certain combinations of materials, requires small layer thicknesses to propagate, as demonstrated for the cases listed in Table 1 (the examples listed in Table 1 refer to leaky Sezawa waves traveling below their cut-off).

According to Figure 6, the IL per wavelength (calculated as IL/N_λ_, for N_λ_ = L/λ) of the Rayleigh and LS waves progresses from −0.58 to −0.07 dB/λ, and from −0.64 to −0.094 dB/λ with increasing ZnO h/λ from 0.05 to 0.45 (having subtracted the 6 dB of bidirectionality). These IL values, even including the conversion loss, are just a little higher than those measured in epitaxial ScAlN thin film grown by molecular beam epitaxy on 4H-SiC [15], or in atomically flat AlN grown onto sapphire [31], single-crystal, and polycrystalline diamond substrates [32], although an epitaxial piezoelectric film is expected to have lower propagation loss than the sputtered polycrystalline one due to the lower dislocation density and roughness of both the free surfaces and interfaces. Despite the large IDT center-to-center distance (6600 µm), the appearance of the Sezawa wave in all the tested devices indicates high crystal quality and high performance of the SAW devices fabricated. The experimental results clearly show that the Sezawa waves have an operating frequency, signal amplitude, and electromechanical coupling coefficient higher than the Rayleigh mode, which is consistent with our calculations. For these reasons, the Sezawa wave is therefore the preferred mode for device applications.

## 5. Conclusions

This work reports numerical simulations and experimental results on the propagation of Rayleigh and leaky-Sezawa waves in ZnO/fused silica substrates. Highly resistive, c-axis oriented piezoelectric ZnO thin films have been grown by the RF reactive magnetron sputtering technique onto fused silica substrates at 200 °C. The SAW propagation properties in ZnO/SiO_2_ have been investigated experimentally for layer thicknesses ranging from 1.8 up to 6.6 µm. Besides the propagation of the fundamental and harmonic Rayleigh modes, Sezawa modes arise due to the slow sound propagation velocity in ZnO compared with that of the substrate. Despite all the tested devices exhibiting layer thickness-to-wavelength ratio values far below the cut-off of the Sezawa mode and despite the very close transverse BAW velocities of the SiO_2_ and ZnO materials, the propagation of low-attenuated leaky longitudinal waves was clearly observed. The experimental results were confirmed by the numerical results obtained through a finite-element analysis which provided us with in-depth insight into the nature of the waves. Such high-frequency modes, identified as leaky-Sezawa (LS) waves under the cut-off, are supposed to be excited by the fundamental and harmonic wavelengths of the IDTs: due to their h/λ values smaller than the ${\left.\frac{h}{\lambda }\right|}_{cut\;off}$, these waves leak energy into the bulk and show a predominant longitudinal character when approaching the velocity of the longitudinal BAW in the substrate. The waves’ propagation in ZnO/fused silica was simulated by using the 2D FEM technique to differentiate the type of the guided surface modes in terms of Rayleigh and LS modes. The LS mode’s velocity is almost double that of the Rayleigh velocity in the substrate, which leads to enhanced operation frequency of the SAW devices without requiring expensive nanofabrication techniques. Although these fast leaky modes have appreciable penetration into the substrate, their transmission amplitudes are high enough for them to be detected by the receiving IDT and have a quite large electromechanical coupling coefficient. A good agreement between the calculated and measured LS wave velocities has been observed, which allowed us to assess the existence of a low-loss region below the cut-off.

Most of the SAW devices are typically fabricated onto bulk single-crystal piezoelectric substrates or thin piezoelectric films deposited on non-piezoelectric substrates (such as Si or sapphire) and include metal (Al or Au) IDTs. They are all high-cost and opaque materials, which prevents their application in transparent devices such as flat-panel displays [33], window electronics [34], and solar cells [35]. Owing to its merits of low cost, good transparency, and easy fabrication, the glass substrate-based SAW UV sensors [26], especially when combined with transparent conductive electrodes [36], can perform various sensing functions (including pressure, temperature, and UV sensing) [16,37] on windows and screens without blocking the visible light.

In the available scientific literature, there are many papers describing the SAWs’ propagation in ZnO/fused silica substrates for sensing, electronic, and optical applications [26,36,37,38,39]. In references [40,41], temperature sensing, humidity, and particle concentration sensing performances have been achieved by using the ZnO/glass-based SAW devices, thus demonstrating the great potential for applications in transparent electronics. Since glass substrates are widely used in optics, biology, photovoltaics, and electronics [40], glass-based SAW devices have great potential to be integrated into these application fields. The scientific novelty of the present paper consists in the experimental and theoretical study of the Sezawa mode propagation in ZnO/fused silica under the cut-off, for ZnO layers with a normalized thickness as thin as 0.023. The experimental investigation performed on several devices with different ZnO layer thicknesses allowed us to demonstrate the existence of a low-loss region under the cut-off, successively confirmed by FEM studies.

The novelty of the present theoretical and experimental study consists in having demonstrated the existence of a low-loss region for the propagation of high-K^2^, high-velocity, low-loss LS modes: by designing IDTs with optimized characteristics, high-frequency SAW devices can be fabricated which are based on ZnO films with thicknesses that we previously thought were only useful for exciting the Rayleigh wave.

The LS waves can be of practical utility if exploited in SAW signal processing devices that operate at elevated frequencies; indeed, these waves appearing in “slow on fast” structures can be an alternative path to further increase the operating frequency of SAW devices. Moreover, the combination of split IDTs with a *slow-on-fast* substrate allows for the fabrication of multi-frequency ZnO/SiO_2_ single device structures which can represent a promising solution for the development of a multi-parameter sensing platform [2,42]; multiple excitation frequencies with different sensing properties can allow for the parallel analysis of the same measurand with improved accuracy.

## Figures and Tables

**Table 1:** The medium where the LS propagates, the film normalized thickness, and the reference.

Propagating Medium	h/λ	Reference
GaN/sapphire(11–20)	0.2125	[7]
GaN/sapphire	0.125–0.875	[7]
AlN(0001)/diamond(111)	0.1875–0.75	[4]
AlN/Diamond	0.15	[5]
AlN/iso-Diamond	*	[5]
AlN/iso-Diamond/c-TiAl	*	[5]
AlN/diamond	0.09	[17]
AlN/4H–SiC(0001)[1–100]	0.3–0.5	[6]
GaN/4H–SiC(0001)[11–20]	~0.7	[6]
Sc_0.09_Al_0.91_N/c-sapphire	0.425	[18]
ZnO/(001)<110>diamond	*	[19]
Iso-Cd/iso-Cr	Appr. 0.08	[20]
Au/fused quartz	0.0328	[21]
a-SiO_2_/128°YX-LiNbO_3_	0.41 and 0.62	[22]
ScAlN/4H-SiC	0.1, 0.12 and 0.17	[15]
GaN/SiC	from 0.2 to 0.48	[23]
ZnO/SiC	~0.1	[24]
ZnO/fused silica	0.023 to 0.45	Present paper

* Theoretical paper.

## Data Availability

The original contributions presented in this study are included in the article. Further inquiries can be addressed to the corresponding author.
